# Effect of New Zealand Blackcurrant Extract on Cycling Performance and Substrate Oxidation in Normobaric Hypoxia in Trained Cyclists

**DOI:** 10.3390/sports7030067

**Published:** 2019-03-16

**Authors:** Mark Elisabeth Theodorus Willems, Mehmet Akif Şahin, Tim Berendsen, Matthew David Cook

**Affiliations:** 1 Institute of Sport, University of Chichester, College Lane, Chichester PO19 6PE, UK; akif@hacettepe.edu.tr (M.A.Ş.); timberendsen91@gmail.com (T.B.); matthew.cook@worc.ac.uk (M.D.C.); 2 Department of Nutrition and Dietetics, Faculty of Health Sciences, Hacettepe University, Sihhiye, 06100 Ankara, Turkey; 3 Human Movement Sciences, Faculty of Health, Medicine and Life Sciences, Maastricht University, 6229 ET Maastricht, The Netherlands; 4 School of Sport and Exercise Science, University of Worcester, Henwick Grove, Worcester WR2 6AJ, UK

**Keywords:** time trial, substrate oxidation, hypoxia, anthocyanins, indirect calorimetry, New Zealand blackcurrant, sports nutrition

## Abstract

New Zealand blackcurrant (NZBC) extract enhanced exercise-induced fat oxidation and 16.1 km cycling time trial (TT) in normobaric normoxia. The effect of NZBC extract on physiological and metabolic responses was examined during steady state cycling and a 16.1 km TT in normobaric hypoxia. This study used a randomized, double-blind, crossover design. Eleven healthy male cyclists (age: 38 ± 11 y, height: 179 ± 4 cm, body mass: 76 ± 8 kg, $\dot{V}$O_2max_: 47 ± 5 mL·kg^−1^·min^−1^, mean ± SD) ingested NZBC extract (600 mg·day^−1^ CurraNZ® containing 210 mg anthocyanins) or a placebo (600 mg microcrystalline cellulose M102) for seven days (washout 14 days) and performed a steady state cycling test (3 × 10 min at 45%, 55% and 65% $\dot{V}$O_2max_) followed by a 16.1 km TT at a simulated altitude of ~2500 meters (~15% of O_2_). Indirect calorimetry was used to measure substrate oxidation during steady state cycling. Intake of NZBC extract had no effect on blood glucose and lactate, heart rate, substrate oxidation, and respiratory exchange ratio during steady state cycling at 45%, 55% and 65% $\dot{V}$O_2max_, and on 16.1 km TT performance (placebo: 1685 ± 92 s, NZBC extract: 1685 ± 99 s, *P* = 0.97). Seven days intake of New Zealand blackcurrant extract does not change exercise-induced metabolic responses and 16.1 km cycling time trial performance for moderately endurance-trained men in normobaric hypoxia.

## 1. Introduction

Blackcurrant is a berry with high nutritional value and many health benefits [1]. The nutritional value and health benefits are obtained from the polyphenol content of the blackcurrant, consisting primarily of the anthocyanins delphinidin-3-*O*-rutinoside, delphinidin-3-*O*-glucoside, cyanidin-3-*O*-rutinoside and cyanidin-3-*O*-glucoside [2]. Anthocyanins provide the blackcurrant with antioxidant and anti-inflammatory effects. Other nutritional ergogenic aids with polyphenol content, such as Montmorency tart cherry, chokeberry and pomegranate have recently provided observations with applications for sport and exercise [3,4,5].

In the case of blackcurrants, a seven-day intake of New Zealand blackcurrant extract improved 16.1 km cycling time trial performance by 2.6% in endurance-trained male cyclists [6]. In addition, the intake of blackcurrant increased blood flow in the forearm after venous occlusion at rest by 22% (i.e., relative to pre-values) during typing [7] and a larger femoral artery diameter by about 6–8% was observed during a 2-min 30% isometric contraction of knee extensors [8]. An increased blood flow may provide better oxygenation of skeletal muscles, as well as improved clearance of muscle metabolites associated with exercise-induced fatigue [9]. In general, vasodilation is associated with a release of nitric oxide (NO) from the endothelium. Blackcurrant juice concentrate activated endothelial NO synthase via the Akt/PI3 kinase pathway in human umbilical vein endothelial cells [10]. In support, Nakamura et al. [11] observed endothelium-dependent relaxation of contracted rat aortic rings with exposure to blackcurrant concentrate. Caution is required to generalize results from in vitro studies, however the high anthocyanin intake in women was associated with lower arterial stiffness and central blood pressure [12], which suggests beneficial cardiovascular effects from regular the intake of anthocyanins.

Many studies have metabolic, physiological and cardiovascular observations with a seven-day intake of New Zealand blackcurrant during exercise and at rest in normobaric normoxic conditions [6,8,13,14,15,16,17]. For example, New Zealand blackcurrant extract enhanced whole-body fat oxidation during cycling in males and females [6,13,16] and increased 16.1 km time-trial performance [6]. However, exercise at altitude affects energy metabolism and fatigue of skeletal muscles [18], for example maximum oxygen uptake ($\dot{V}$O_2max_) and power output decrease with altitude. The physiological mechanism behind the decrease in exercise performance at altitude depends mainly on the lower partial pressure of oxygen. A larger role for metabolic processes that lead to the accumulation of fatigue related metabolites (i.e., ADP, Pi and H^+^) may contribute to reduced exercise performance in hypoxic environments [18]. In addition, exercise in a hypoxic environment may provide higher levels of oxidative stress [19] and affect exercise-induced fatigue, with blackcurrant having the ability to reduce exercise-induced oxidative stress [20]. The hypoxia-induced fatigue mechanisms may be attenuated by the intake of blackcurrant. The effects of an anthocyanin-rich berry supplement on metabolic, physiological and performance responses during cycling at altitude is not known.

Therefore, the primary aim of the present study was to examine the effect of New Zealand blackcurrant extract on the 16.1 km cycling time-trial performance in normobaric hypoxia. This study also examined whether there was an effect of New Zealand blackcurrant extract on the physiological and metabolic responses during low and moderate intensity cycling in normobaric hypoxia.

## 2. Materials and Methods

### 2.1. Participants and Study Design

Eleven healthy male cyclists and triathletes between the ages of 18 and 55 years provided written informed consent for voluntary participation in the study. Participants were recruited from local triathlon and cycling clubs and their characteristics are presented in Table 1. Participants were required to have at least three years membership, were not involved in a training program, cycled for 8–10 h per week and were able to complete the 16.1 km cycling time trial in less than 40 min. None of the participants were required to have a washout period because of taking other supplements. The University of Chichester Research Ethics Committee approved the study (approval number: 1819_06). All procedures were conducted in accordance with the 2013 Declaration of Helsinki. Participants did not receive payment for taking part in the study.

A cross-over, randomized, double-blind design was used with a 2-week washout period separating the 7-day supplementation periods. A 2-week washout period returned biochemical parameters and biomarkers of antioxidant status and oxidative stress from 500 g 1-month strawberry intake to baseline [21]. All testing took place in a temperature controlled (15 °C) normobaric hypoxic chamber set at a simulated altitude of ~2500 m (15% O_2_) (TIS Services, Medstead, United Kingdom). Participants visited the laboratory three times, one screening and familiarization visit and two visits for testing after 7-day intake of the placebo or New Zealand blackcurrant extract. For each visit, the participants were instructed to arrive in a rested and hydrated state, at a similar time in the morning and at least 2-hour postprandial after consuming a breakfast consisting of one slice of bread and a glass of water [6]. Participants were instructed to abstain from intense exercise for 48 h and alcohol for 24 h before testing. Cycling was performed on the SRM (Schoberer Rad Meßtechnik) ergometer (SRM International, Jülich, Germany). Participants used their own cycling shoes and pedals. During the first visit, settings of the saddle height and setback, along with handlebar reach and drop, were adjusted by the participant and replicated during subsequent visits. Heart rate was continuously measured during all tests (Polar RS 400, Polar Electro Oy, Kempele, Finland).

### 2.2. Screening and Familiarization Visit

During the first visit, height (Seca 213, Seca, Birmingham, United Kingdom), body mass (Kern ITB, Kern, Germany) and body fat (Tanita BC418, segmental body composition analyzer, Tanita, Illinois, USA) were measured. Following anthropometrical measurements, the participants performed an incremental cycling protocol to record blood lactate values and stopped when lactate exceeded 2 mmol·L^−1^. After a rest period of 10 min, an incremental cycling protocol was performed to determine maximum oxygen uptake ($\dot{V}$O_2max_). Following another rest period of 20 min, the participants practiced the 16.1 km (i.e., 10 miles) cycling time trial.

### 2.3. Experimental Visits

Before visits 2 and 3, participants ingested two capsules of New Zealand blackcurrant extract for seven days (one capsule containing 300 mg active cassis, of which 105 mg were anthocyanins, i.e., 35–50% delphinidin-3-*O*-rutinoside, 5–20% delphinidin-3-*O*-glucoside, 30–45% cyanidin-3-*O*-rutinoside, 3–10 % cyanidin-3-*O*-glucoside) (CurraNZ^TM^, Health Currancy Ltd., Surrey, United Kingdom), or identical looking placebo capsules (2 × 300 mg microcrystalline cellulose M102). In the first six days of dosing, it was recommended to have capsule intake with an 8 h interval in between, with the last two capsules taken on the testing day with the recommended breakfast. In the second and third visit, blood pressure in rest was taken four times using an automated cuff (OMRON 705 IT, Medisave, Weymouth, United Kingdom) and in normobaric normoxic conditions, with the last three measurements averaged to obtain mean blood pressure. Arterial oxygen saturation was measured after 10 min acclimation to hypoxia with a pulse oximeter (ChoiceMMed, MD300C41, Medisave, Weymouth, United Kingdom). Subsequently, participants performed 3 bouts of 10 min cycling at 45%, 55% and 65% $\dot{V}$O_2max_ with expired air collected in the final 2 min of each bout. Following a 20 min rest period, participants performed a 16.1 km cycling time trial.

### 2.4. Dietary and Training Restrictions

Participants completed a food diary 48 h before the second visit. Participants were instructed to replicate this food diary before the third visit. Food diaries were analyzed for carbohydrate, fat and protein intake (Nutritics LTD., Dublin, Ireland). Participants reported adherence to the supplement intake for both conditions and to the food diary for the third visit. Carbohydrate, fat and protein intakes were similar for the placebo and New Zealand blackcurrant extract conditions (P > 0.05) (Table 2).

### 2.5. Incremental Cycling Test for Oxygen-Cycling Power Relationship

The incremental cycling protocol in visit one was performed to determine the relationship between oxygen uptake and power output. Participants cycled stages of 4.5 min starting at 80 W with stage increments of 30 W [22] and without rest periods in between stages. During the last minute of each stage, expired air was collected by using the Douglas bag method (Cranlea & Co. Bourneville, Birmingham, United Kingdom). Furthermore, a capillary blood sample was taken from the finger in the last 30 seconds of each stage. Blood samples were analyzed for lactate levels (YSI 2300 STAT PlusTM analyzer, YSI Life Sciences, Yellow Springs, USA) and the incremental cycling test was terminated when blood lactate exceeded 2 mmol·L^−1^.

### 2.6. Incremental Cycling Test for Maximum Oxygen Uptake

The incremental cycling protocol started at a power of 50 W for 4 minutes with increments of 30 W every minute until volitional exhaustion [6]. Participants were allowed to cycle at a self-selected pedal rate between 70 and 90 rpm. Expired gas was collected in samples of at least 45 seconds by using the Douglas bag method. A minimum collection sample of at least 30 seconds was required for analysis of the last expired air collection. The gas samples were analyzed for fractions of expired oxygen (O_2_) and carbon dioxide (CO_2_) using a gas analyzer (Series 1400, Servomex, Crowborough, United Kingdom), calibrated by using known gases (Linde Gas UK Ltd., West Bromwich, United Kingdom). Volumes of the expired gases were measured by using a dry gas meter (Harvard Apparatus Ltd., Edenbridge, United Kingdom). Capillary blood samples from the finger were taken 3 minutes after the end of the test for lactate analysis. Participants reached their $\dot{V}$O_2max_ if they met at least two of the following criteria; (1) an increase in $\dot{V}$O_2_ of <2.1 mL∙kg^−1^∙min^−1^ between the last 2 expired gas collections, (2) plasma lactate > 5.5 mmol∙L^−1^, (3) respiratory exchange ratio (RER) >1.15 [23], or (4) a heart rate within 10 beats·min^−1^ from the age-predicted maximum heart rate (age-predicted max HR = 207 − 0.7 × age).

### 2.7. Submaximal Cycling at 45%, 55% and 65% of $\dot{V}$O_2max_

The values of the lactate threshold test were used to calculate the power of the submaximal cycling test. Participants had to cycle at 45%, 55% and 65% of $\dot{V}$O_2max_ for 10 min at each intensity. Expired gases were collected with the Douglas bag technique during minutes 8–10 of each submaximal intensity and analyzed for respiratory parameters. Carbohydrate and fat oxidation rates were calculated with equations from Jeukendrup and Wallis [24]. The following calculations were used to calculate carbohydrate and fat oxidation, where protein oxidation was assumed to be negligible:

(1) $$\mathrm{Fat}\;\mathrm{oxidation}=1.695\times \dot{V}O_{2}-1.701\times \dot{V}{\mathrm{CO}}_{2}$$

(2) $$\mathrm{Low}\;\mathrm{intensity}\;(45\%\;\dot{V}O_{2\max})\;\mathrm{carbohydrate}\;\mathrm{oxidation}=4.344\times \dot{V}{\mathrm{CO}}_{2}-3.061\times \dot{V}O_{2}$$

(3) $$\mathrm{Moderate}\;\mathrm{intensity}\;(55\%,\;65\%\;\dot{V}O_{2\max})\;\mathrm{carbohydrate}\;\mathrm{oxidation}=4.210\times \dot{V}{\mathrm{CO}}_{2}-2.962\times \dot{V}O_{2}$$

### 2.8. Cycling Time Trial—16.1 km (i.e., 10 mile)

Participants were instructed to cycle at a self-selected gear and pedal rate to complete the cycling time trial in the fastest time possible. Feedback was received at 25%, 50% and 75% of the distance covered, and no encouragement was provided. Testing conditions (e.g., first requested fan use) during the time trial were replicated between subsequent time trial tests.

### 2.9. Statistical Analysis

A third-degree polynomial was used to describe the relationship between the power during cycling and blood lactate [25]. Statistical analyses were executed with IBM SPSS statistical software version 22 (IBM, Armonk, USA). Values are reported as mean ± SD. All parameters were tested for normal distribution and homogeneity with the Shapiro–Wilk test. The paired samples two-tailed t-test was used to analyze the metabolic and physiological responses at 45%, 55% and 65% of $\dot{V}$O_2max_, cycling time trial performance and 24 h dietary measures. A significance level α of 0.05 was used for all statistical tests.

## 3. Results

### 3.1. Blood Pressure and Arterial Oxygen Saturation at Rest

There were no differences between conditions for systolic (NZBC: 128 ± 14, Placebo: 130 ± 13 mmHg, *P* = 0.176) and diastolic blood pressure (NZBC: 77 ± 10, Placebo: 77 ± 8 mmHg, *P* = 0.673) in normobaric normoxia. Arterial oxygen saturation after 10 min at normobaric hypoxia showed no difference between conditions (NZBC: 93 ± 2 %, Placebo: 95 ± 3 %, *P* = 0.308) (Table 3).

### 3.2. Responses to Submaximal Cycling

NZBC extract had no effect on $\dot{V}$O_2_, $\dot{V}$CO_2_, cycling economy, glucose, lactate, carbohydrate oxidation, fat oxidation, RER and heart rate at each of the exercise intensities, i.e., 45% (117 ± 26 W), 55% (161 ± 29 W) and 65% of $\dot{V}$O_2max_ (205 ± 33 W) (Table 3).

### 3.3. Performance of the 16.1 km Cycling Time Trial

No difference was observed in the time to complete the 16.1 km cycling time trial between NZBC extract and the placebo in normobaric hypoxia (NZBC: 1685 ± 99 s; Placebo: 1685 ± 92 s, *P* = 0.974).

## 4. Discussion

The present study provides observations on the effect of seven days intake of New Zealand blackcurrant extract on physiological responses and 16.1 km time trial performances of moderately trained male cyclists in normobaric hypoxia (~15%, ~2500 m). No changes were observed for physiological and metabolic responses, i.e., heart rate, minute ventilation, oxygen uptake, respiratory exchange ratio and substrate oxidation at the three different cycling intensities, i.e., 45%, 55% and 65% of $\dot{V}$O_2max_. In addition, the 16.1 km cycling time trial performance was not affected. Peripheral oxygen saturation in normobaric hypoxia was similar for the placebo and NZBC extract conditions. If there had been an increase in blood flow due to blackcurrant intake [7,8] in normobaric hypoxia, the peripheral oxygen saturation may have been higher after blackcurrant intake, however this was not the case. Peripheral oxygen saturation at ~2500 m was similar to Morishima et al. [26] at 2700 m. The absence of a change in peripheral oxygen saturation observations with the intake of New Zealand blackcurrant extract agrees with Muggeridge et al. [27] and MacLeod et al. [28] in normobaric hypoxia (~2500 m) after beetroot juice intake. However, Masschelein et al. [29] observed a higher peripheral oxygen saturation at rest in severe hypoxia (11% O_2_) compared to the control group after nitrate intake. In that study, peripheral oxygen saturation dropped to lower values (~72–74%) during exercise. A limitation of this study was that peripheral oxygen saturation was not measured during exercise.

### 4.1. Metabolic Responses

In the present study, a seven-day intake of New Zealand blackcurrant extract had no effect on fat and carbohydrate oxidation rates during steady state cycling in normobaric hypoxia. These observations are in contrast with previous studies on the effects of New Zealand blackcurrant extract on fat and carbohydrate oxidation during cycling in normobaric normoxia in endurance-trained males [6,13]. For example, a higher fat oxidation of 27% was observed during cycling at 65% $\dot{V}$O_2max_, with a trend (P = 0.077) for higher fat oxidation at 45% $\dot{V}$O_2max_ [6]. At moderate altitudes during exercise, carbohydrate oxidation is higher (~2000 m [30], ~2700 m [26]) and associated with higher levels of epinephrine and norepinephrine [30]. The hormonal and metabolic response to exercise at normobaric hypoxia may constitute a strong stimulus for carbohydrate oxidation that cannot be overcome by the ability of New Zealand blackcurrant extract to enhance fat oxidation as observed in normobaric normoxic conditions [6]. In addition, previous studies have suggested that vasodilation by blackcurrant intake may partly explain substrate oxidation and performance effects. However, hypoxia-induced compensatory vasodilation [31,32] may limit or even prevent the potential mechanisms for the effects of New Zealand blackcurrant extract. As far as it is known, there are no other studies that have examined the responses by supplementation on exercise-induced substrate oxidation at normobaric hypoxia.

### 4.2. Time-Trial Performance (16.1. km)

New Zealand blackcurrant extract did not provide a performance enhancing effect in the 16.1 km cycling time trial at normobaric hypoxic conditions, in contrast with Cook et al. [6] in normobaric normoxic conditions who observed an increase of 2.4%. The absence of enhanced fat oxidation and no effect on 16.1 km cycling time trial performance in the present study contradict the association of enhanced fat oxidation and performance enhancement in normobaric normoxic conditions [6]. In Cook et al. [6], the dose of anthocyanins was even half (~105 mg anthocyanins) of that used in the present study (~210 mg anthocyanins).

An acute intake of beetroot juice also did not affect a 10-km time trial in normobaric hypoxia (~ 2500 m) in trained male cyclists [28]. In contrast, enhanced performance by 2.2% for a 16.1 km time trial was observed with an acute intake of beetroot juice [27]. In Muggeridge et al. [27], the 16.1 km time trial by nine participants was completed in 1702 ± 15 s (mean ± SEM). In the present study with 11 participants, the baseline 16.1 km time trial was somewhat faster but completed with more variation, i.e., 1685 ± 28 s (mean ± SEM). This may also suggest more variability in the training status of participants. Previous studies have shown that nitrate supplements have a reduced effect on well-trained athletes [33] and well-trained athletes have a greater presence of nitric oxide synthase [34]. Since anthocyanins in blackcurrant extract seem to induce an increased production of endothelial-derived nitric oxide [11], it was thought that blackcurrant would have a similar effect as nitrate supplements and therefore lead to a higher vasodilation and thus blood flow, even in well-trained athletes. However, this does not seem to be supported by observations in the present study. An interesting aim for future research would be to investigate the effect of New Zealand blackcurrant extract in cyclists with very different training status and/or cycling ability.

## 5. Conclusions

It is concluded that New Zealand blackcurrant does not have an effect on 16.1 km time trial performance, metabolic responses and physiological responses during cycling in normobaric hypoxia (~2500 m). These findings do not support New Zealand blackcurrant as an ergogenic aid for endurance performance in hypoxia.

## Figures and Tables

**Table 1:** Participant characteristics (*n* = 11).

Age (years)	38 ± 11
Height (cm)	179 ± 4
Body mass (kg)	76 ± 8
$\dot{V}$O_2max_ (mL∙kg∙min^−1^)	47 ± 7
RER_max_	1.24 ± 0.07
Power (lactate 2 mmol∙L^−1^) (W)	252 ± 38
Lactate_max_ (mmol∙L^−1^)	7.8 ± 2.9
HR_max_ (beats∙min^−1^)	174 ± 18
WRmax (W)	398 ± 38
Body fat (%)	15.5 ± 5.9

Data are reported as mean ± SD.

**Table 2:** Dietary daily intake for placebo and New Zealand blackcurrant (NZBC) extract conditions.

	Placebo	NZBC Extract
Carbohydrate (g)	221 ± 52	232 ± 56
Carbohydrate (g·kg body mass^−1^)	3.00 ± 0.78	3.18 ± 0.93
Fat (g)	65 ± 27	73 ± 30
Fat (g·kg body mass^−1^)	0.89 ± 0.40	1.00 ± 0.44
Protein (g)	95 ± 35	91 ± 30
Protein (g·kg body mass^−1^)	1.29 ± 0.53	1.24 ± 0.45

**Table 3:** Responses during cycling at low (45% $\dot{V}$O_2max_) and moderate (55% and 65% $\dot{V}$ O_2max_) intensities for placebo and New Zealand blackcurrant (NZBC) extract conditions.

	$45\%\;\dot{V}O_{2\max}$		$55\%\;\dot{V}O_{2\max}$		$65\%\;\dot{V}O_{2\max}$	
Parameter	Placebo	NZBC	Placebo	NZBC	Placebo	NZBC
$\dot{V}$O_2_ (L·min^−1^)	1.58 ± 0.18	1.59 ± 0.17	1.97 ± 0.20	2.00 ± 0.19	2.38 ± 0.21	2.40 ± 0.23
$\dot{V}$CO_2_ (L·min^−1^)	1.45 ± 0.19	1.48 ± 0.13	1.83 ± 0.20	1.88 ± 0.15	2.23 ± 0.23	2.28 ± 0.20
Relative intensity ($\dot{V}$O_2max_)	44 ± 3	44 ± 4	54 ± 4	56 ± 4	66 ± 4	67 ± 4
Economy (mL·kg^−1^·W^−1^)	10.9 ± 1.4	11.1 ± 1.7	9.8 ± 1.1	10.1 ± 1.5	9.3 ± 1.1	9.4 ± 1.3
Heart rate (beats·min^−1^)	101 ± 9	103 ± 13	116 ± 11	116 ± 13	133 ± 12	132 ± 13
Lactate (mmol·L^−1^)	1.06 ± 0.36	1.03 ± 0.27	1.03 ± 0.28	1.04 ± 0.31	1.37 ± 0.45	1.56 ± 0.57
Glucose (mmol·L^−1^)	4.07 ± 0.50	4.23 ± 0.44	4.15 ± 0.17	3.84 ± 0.67	4.05 ± 0.34	4.05 ± 0.39
CHox (g·min^−1^)	1.47 ± 0.36	1.55 ± 0.19	1.90 ± 0.34	2.00 ± 0.27	2.34 ± 0.42	2.48 ± 0.35
FATox (g·min^−1^)	0.21 ± 0.11	0.18 ± 0.10	0.21 ± 0.09	0.19 ± 0.14	0.24 ± 0.12	0.20 ± 0.16
Respiratory exchange ratio	0.92 ± 0.04	0.93 ± 0.03	0.93 ± 0.03	0.94 ± 0.04	0.94 ± 0.04	0.94 ±0.05

Data were collected after 7 days intake of either NZBC extract or placebo during 10-min cycling bouts, and 2 h post-prandial after a low-calorie breakfast (1 slice of bread and the last capsules). CH_ox_, carbohydrate oxidation; FAT_ox_, fat oxidation. Data reported as mean ± SD from 11 participants.
